# Bispectral Index Monitoring in Patients Undergoing Open Heart Surgery

**DOI:** 10.5935/1678-9741.20160038

**Published:** 2016

**Authors:** Hanife Karakaya Kabukcu, Nursel Sahin, Kezban Ozkaloglu, Ilhan Golbasi, Tulin Aydogdu Titiz

**Affiliations:** 1 Akdeniz University Medical Faculty, Antalya, Turkey

**Keywords:** Cardiopulmonary Bypass, Anesthesia, Monitoring, Intraoperative

## Abstract

**Introduction:**

To obtain the optimal anesthesia depth is not easy in cardiovascular surgery patients where the haemodynamic reserve is limited, due to reasons such as not being able to give the desired dose of anesthetic agent, or the change in the pharmacokinetics of the agent in the heart-lung machine. This study was planned to assess the contribution of bispectral index (BIS) monitoring in the depth of anesthesia.

**Methods:**

The patients were divided into 2 groups, and BIS monitoring was used for each patient. Group 1 (G1 n=35): keeping the BIS monitor screen open, the anesthesia need was set. Group 2 (G2 n=35): BIS monitor was tied to the patient and the monitor screen was closed in such a way that the anaesthesist couldn't see the BIS value. When the recording time came, the data on the monitor was recorded. The need for the anesthetic agent was set according to the parameters such as haemodynamics or follow up of pupils, instead of BIS value, by titrating the anesthetic infusion doses.

**Results:**

BIS values were similar in both groups before the induction, BIS values in both groups showed a decrease, showing no significant statistical difference (*P*>0.05). One patient in each group said that he dreamt, and one patient in G2 said that he had heard a noise and felt that he was taken from one place to another.

**Conclusion:**

The management should be done with clinical evaluation, haemodynamics and other monitorization methods and BIS monitoring findings together.

**Table:** 

Abbreviations, acronyms & symbols	
BIS	= Bispectral index
CABG	= Coronary artery bypass graft
CVP	= Central venous pressure
DAP	= Diastolic arterial pressure
ECG	= Electrocardiography
EEG	= Electroencephalography
EF	= Ejection fraction
ETAG	= End-tidal anaesthetic gas
EtCO_2_	= End-tidal CO_2_
HR	= Heart rate
MAP	= Mean arterial pressure
PET	= Positron emission tomography
SAP	= Systolic arterial pressure
SpO_2_	= Oxygen saturation
TIVA	= Total intravenous anaesthesia
TOF	= Train-of-four

## INTRODUCTION

Adjustment of the depth of anesthesia in open heart surgery is difficult due to many factors. Deep anesthesia may cause hypotension and circulatory insufficiency^[1]^, and for the risk of haemodynamic disturbance, keeping the anesthesia superficial may increase the risk of awakening in this patient group with a restricted haemodynamic reserve. Besides, during the heart-lung machine use, the pharmacokinetics of anesthetic drugs may change. For this reason, the same dose of drug used in different patients may be less or more^[2,3]^. As a result, it is difficult to control the anesthesia depth in some patients having open heart surgery, and new monitorization modalities are needed in addition to the conventional clinical data of these patients^[4]^.

Respiratory and haemodynamic monitoring in the anesthesia practice for cardiac surgery is considerably advanced, but there are limited modalities for brain function monitoring. BIS monitoring is a modality developed as a result of studies that aimed to assess the electroencephalography (EEG) in a simpler and more reliable manner, which digitizes the brain's electrical activity. The BIS index shows the cerebral metabolic activity. The parallelism between the BIS value and the brain glucose usage was shown by positron emission tomography (PET) studies^[5,6]^. The use of BIS to assess the anesthesia depth is recommended because it shows the electrical activity in the deep cortical brain layers^[7,8]^.

There are few studies that examine the use of BIS monitoring in cardiac anesthesia^[9]^. In cardiac anaesthesia, both total intravenous anesthesia (TIVA) and inhaled anaesthesia were used. BIS correlates well with measured plasma concentrations of propofol, and end-tidal anaesthetic gas (ETAG) concentrations. ETAG monitoring protocol with alarms set at 0.7-1.3 MAC reduced the incidence of awareness. TIVA is often regarded as an independent risk factor for awareness because of the risk of interruption of drug supply, and the lack of an equivalent measure to ETAG^[10]^.

The aim of this study is to study the effect of BIS monitoring on anesthesia depth, the use of anesthetic, analgesic and inotropic drugs, and intraoperative awareness and awakeness in patients on whom moderate hypothermia was applied to in open heart surgery.

## METHODS

A total of 70 patients that will have open heart surgery were included in the study with the approval of the hospital's ethics committee. Patients who needed emergency surgery, had unstable angina pectoris history, hemodynamic instability, organ transplants, acute myocardial infarction, acute heart failure, preoperative medical and mechanical inotropic support, pulmonary embolism, renal failure and hepatic cirrhosis history were excluded from the study.

All the patients were underwent the coronary artery bypass graft (CABG) operation. The patients were divided into 2 groups, and BIS monitoring was used for each patient. BIS monitor (Aspect Medical Systems) probe was placed on the patients' foreheads and the basal values were recorded. Group 1 (G1 n=35): keeping the BIS monitor screen open, the anesthesia need was set. Group 2 (G2 n=35): BIS monitor was tied to the patient and the monitor screen was closed in such a way that the anesthesist couldn't see the BIS value. When the recording time came, the data on the monitor was recorded. The need for the anesthetic agent was set according to the parameters such as haemodynamics or follow up of pupils, instead of BIS value, by titrating the anesthetic infusion doses. Electrocardiography (ECG), invasive arterial pressure, central venous pressure (CVP), oxygen saturation (SpO_2_), End-tidal CO_2_ (EtCO_2_), peripheral nerve stimulator train-of-four (TOF), and BIS monitoring were performed in all of the patients.

For the induction of anesthesia, 4-5 µg/kg fentanyl, 0.2 mg/kg etomidate and 0.1 mg/kg vecuronium were used and the patient was intubated. Maintenance of the anesthesia was done by 4-8 mg/kg/hour propofol and 15 µg/kg/hour remifentanil. Propofol and remifentanil infusion doses were titrated to keep the BIS value between 35-45% during the operation. The haemodynamic data values were recorded preoperatively, after induction, at the time of skin incision, after sternotomy, during bypass, at the time of disconnection from the pump, and at the end of the operation.

All the patients were subject to moderate hypothermia (28-32ºC), and membrane oxygenators were used. After cardiac cross-clamp application perfusionist initiates delivery of 10 ml/kg cardioplegia by mixing oxygenated blood with a crystalloid solution (plegisol) at a ratio of 4:1. During aortic cross-clamping, multidose cold blood cardioplegia is applied to maintain cardioplegic arrest and myocardial hypothermia. Just before aortic unclamping normothermic blood cardioplegia was applied. At the end of the operation, the patients were monitored and transferred to the cardiovascular surgery intensive care unit on mechanical ventilation support.

One day following the operation, the patients were questioned about awakeness and awareness.

### The questions for the Awareness Score were modified from Sebel^[11]^

1. What is the last thing you remember before you fell asleep?
2. What is the first thing you remembered when you woke up?
3. Do you have any memories between these two events?
4. Did you dream during anesthesia and surgery?
5. What is the most unpleasant thought you had during the intervention?

### Ethical Issues

All procedures performed in studies involving human participants were in accordance with the ethical standards of the institutional and/or national research committee and with the 1964 Helsinki declaration and its later amendments or comparable ethical standards.

Informed consent was obtained from all individual participants included in the study.

### Statistical Analysis

Observed data were entered into Microsoft Excel Workbook and analyzed using the SPSS 22 for Windows. Numerical data were analyzed using the independent sample t-test. Paired t-test was used for in-group comparison and unpaired t-test was used for comparison between groups. The categorical data were analyzed using the Chi-square test. A *P*<0.05 was taken to be of statistical significance. Datas were presented as mean ± standard deviation.

## RESULTS

No statistically significant difference was found between the two groups, regarding the preoperative demographic characteristics such as age, weight, height and ejection fraction of the cases. Both groups had the same anesthesia, operation time, and cross-clamp time (*P*>0.05, Table 1).

**Table 1:** Patient characteristics and operative details for groups.

	Group 1	Group 2
Age (year)	58.7±11.4	56.2±10.4
Body height (cm)	166.9±7.9	166.1±7.8
Body weight (kg)	76.0±9.9	75.0±13.5
Ejection fraction (%)	60.2±6.9	59.7±7.9
Operation time (min)	228.3±59.0	248.1±35.9
Anesthesia time (min)	267.4±54.7	289.1±41.4
Cross-clamping time (min)	77.4±26.8	69.1±22.1
Number of anasthomoses	2.9±0.86	2.6±0.4

Values are expressed as mean ± standard deviation

### Heart Rate (HR)

The HRs were similar in both groups before the induction (76.6±14.1 beats/min in G1; 75.0±13.81 beats/min in G2) (Table 2). At the time of skin incision and sternotomy, the values were found to be less than the preoperative values (*P*<0.05). In G1, the HR was fast after the aorta cannula removal and until the end of the operation (*P*<0.05). In G2, there was no statistically significant difference (*P*>0.05). Intergroup comparison was statistically significant at the time of skin incision, after the thorax was closed and at the end of the operation (*P*<0.05).

**Table 2:** Perioperative hemodynamic data.

		Preoperative	Skin incision	After Sternotomy	After pericardiotomy	Before Bypass	Cross-clamp time	After Cross-clamp	After pump	After Aorta cannula removal	Thorax closed	End ofthe operation
HR (cerebrovascular accident/min)	G1	76.6±14.1	66.0±13.3*+	69.9±13.0*	75.2±14.5	60.2±28.3	23±28.6	76.2±29.4	83.7±24.1	86.0±15.6*	84.1±13.4*+	82.8±13.1*+
	G2	75.0±13.8	58.6±14.0*	65.2±12.2*	71.9±12.8	61.0±25.7*	45.0±42.3	69.2±33.3	78.5±13.7	79.9±15.7	75.1±14.2	74.2±13.3
SAP (mmHg)	G1	130.4±22.7	107.6±26. 9*	116.7±18.0	103.7±24. 4*	75.4±15.2	68.6±15. 1*	77.5±27. 3*	96.4±22. 3*	110.9±13. 3*	107.7±16.4*	109.7±18. 2*
	G2	140.3±23.6	117.2±21.4*	112.1±19.5*	110.6±15.7*	75.7±23.7*	59.2±11.4*	72.6±17.8*	98.8±20.6*	113.4±12.2*	108.2±12.7*	109.1±12.5*
MAP (mmHg)	G1	89.8±16.6	77.0±21.1*	85.0±15.3	70.9±14.4*	65.9±13. 7*	652±16.5*+	60.7±17.4*	65.1±16.9*	71.2±11.1*	71.5±14.1*	71.5±13.1*
	G2	91.7±15.4	76.6±15.1*	81.8±14.3	74.6±11.7*	62.0±17.1*	56.3±11.4*	61.3±14.1*	70.3±11.1*	75.6±8.4*	73.1±10.6*	73.4±9.0*
DAP (mmHg)	G1	65.1±13.0	61.4±13.5	62.4±15.5	55.0±13.2*	61.1±15.1	61.9±15.5	53.5±13.2*	54.6±12.3*	53.8±10.3*	52.6±10.8*	53.4±9.1*
	G2	65.0±12.2	59.6±14.4*	65.1±13.5	57.5±11.7*	54.7±16.7*	54.3±12.1*	55.9±12.7*	56.3±9.4*	57.8±9.5*	55.9±7.4*	55.1±7.2*
MCVP (mmHg)	G1		4.0±3.4	3.4±2.9*	2.3±1.7*	1.7±2.3*	1.7±2.4*	3.6±3.4+	5.2±4.1	5.7±4.1	5.3±3.3	5.5±3.3
	G2		5.0±4.5	3.4±4.5*	2.5±4.2*	2.8±4.2*	3.3±4.2*	5.4±3.2*	6.0±3.3*	5.0±4.2*	5.1±2.9*	4.9±2.5*
EtCO_2_ %	G1		23.6±3.6	24.1±4.2	22.0±3.4*	13.2±7.1*	10.0±7.1*	11.1±8.3*	15.8±7.3*	20.9±3.8*	21.9±3.5*	23.4±9.0
	G2		24.4±3.0	25.2±3.3	23.7±3.1*	11.3±8.0*	9.4±7.4*	10.4±9.8*	17.6±6.5*	20.9±3.8*	21.9±3.3*	22.2±2.9*
SpO2 %	G1	97.0±2.1	99.7±0.5*	99.8±0.4*	99.9±0.3*	99.8±0.3	99.7±0.9*	99.7±0.9*	99.9±0.1*	100.0±0.0*+	99.8±0.4*	99.7±0.5*
	G2	97.8±1.4	99.7±0.5	99.7±0.7	99.5±0.4*	99.7±0.4	99.6±1.0	98.5±0.9	99.8±0.6	99.8±0.3	99.9±0.2	99.9±0.2
BiS %	G1	97.1±1.5	45.7±8.5*	44.3±7.1*	39.7±7.8*	38.9±11.9*	43.6±19.9*	31.5±10.0*	36.2±10.0*	37.3±14.0*	38.9±10.2*	37.4±5.7*
	G2	97.4±1.3	41.9±9.3*	40.9±7.8*	36.7±7.5*	35.5±13.5*	39.7±17.7*	25.2±9.1	32.1±11.1*	35.1±10.2*	36.5±11.0*	34.7±8.3*

Values are mean scores ± standard deviation.

* means statistically significant (*P*<0.05), in group comparisons were made in values labelled with *and preoperative values,

+ in the comparison between the groups.

### Systolic (SAP), Diastolic (DAP) and Mean (MAP) Arterial Pressure

No difference was found between G1 and G2 before induction in SAP (130.4±22.7 mmHg in G1; 140.3±23.6 mmHg in G2; *P*>0.05), DAP (65.1±13.0 mmHg in G1; 65.0±12.2 mmHg G2; *P*>0.05) and MAP (89.8±16.6 mmHg in G1; 91.7±15.4 mmHg in G2; *P*>0.05). SAP, DAP and MAP courses were lower during the operation, compared to preoperative values in G1 and G2 (*P*>0.05, Table 2). Intergroup statistical comparison showed significantly high MAP in G1 after cross-clamp removal. No significant difference was detected in the other periods of the operation (*P*>0.05).

### Central Venous Pressure (CVP)

There was no difference between G1 and G2 at skin incision time CVP values (4.0±3.4 mmHg in G1; 5.0±4.5 mmHg in G2; *P*>0.05, Table 2). After the cross-clamp removal, the CVP difference in G1 was significantly lower than in G2 (3.6±3.4 mmHg in G1; 5.4±3.2 mmHg in G2; *P*<0.05). In G1, a decrease was detected in CVP until the cross-clamp was placed (*P*<0.05). No difference was detected until the end of the operation following the aortic clamp placement (*P*>0.05). The CVP values at the skin incision time in G2 coursed low until the aortic clamp was placed, and coursed high afterwards during the operation (*P*<0.05).

### Peripheral Oxygen Saturation (SpO_2_) and End-tidal CO_2_ (EtCO_2_)

The saturation values were in adequate ranges during the whole operation, showing no statistically significant differences between the groups (*P*>0.05). EtCO_2_ values were similar in intergroup comparison (*P*>0.05, Table 2).

### Bispectral Index (BIS)

BIS values were similar in both groups before the induction (97.1±1.5 in G1; 97.4±1.3 in G2; *P*>0.05, Table 2). With induction, BIS values in both groups showed a decrease, without significant statistical difference (*P*>0.05).

### Anesthetic Drug Requirement

Intergroup comparison showed no difference in propofol use during the bypass, after the bypass and in the total dose of the drug (*P*>0.05). Fentanyl use was similar in both groups. The total remifentanil dose was found to be higher in G1 than in G2 (*P*<0.05). The used vecuronium dose was similar in both groups (*P*>0.05). There was no statistically significant difference between the inotropic and vasodilator agents (*P*>0.05). No statistical difference was found in ventilator parameters and blood gas values (*P*>0.05).

### Intraoperative Awareness

When the patients were questioned for intraoperative awareness and awakeness in the postoperative period, one patient in each group said that he dreamt, and one patient in G2 said that he had heard a noise and felt that he was taken from one place to another.

## DISCUSSION

One of the purposes of general anesthesia is to make the patient unconscious, leave the patient unaware of the events during the operation and make him not to remember them in the postoperative period. Awareness is the patient's being awake during the operation, having a bad dream, or remembering some major events^[12]^. Especially in some general anesthesia applications where muscle relaxants are used, 0.9-7% of awareness is reported^[13]^. The patient cannot express this situation at the time of operation, but only after the operation. Awareness or remembering events during the operation may cause postoperative anxiety, sleep disorders, nightmares or posttraumatic stress disorders^[14]^.

Awareness during anesthesia may differ according to the type and depth of anesthesia. Awareness rate is higher in situations where superficial anesthesia is used. To obtain the optimal anesthesia depth is not easy in cardiovascular surgery patients where the haemodynamic reserve is limited, due to reasons such as not being able to give the desired dose of anesthetic agent, or the change in the pharmacokinetics of the agent in the heart-lung machine. The awareness rate is reported between 0.3-4% in cardiovascular surgery^[15,16]^. For this reason, the search for a new monitorization method to assess the depth of anesthesia in cardiovascular surgery exists. This study was planned to assess the contribution of BIS monitoring in the depth of anesthesia.

We reported a rapid decrease in BIS values with anesthesia induction. The BIS recording device assesses the EEG signals in the last 5-10 seconds and it renews the value every second, which allows detection of changes in the brain metabolism in 5-10 seconds. In our study, the BIS values changed between 35-45 during the operation. Especially, the BIS values at the heart-lung pump are low in both groups. The BIS values represent the brain metabolism, the decrease in this value is related to the anesthetic agents' doses, as well as to the other factors affecting the metabolism. Previous studies have reported that to keep the BIS values under 60 provides adequate hypnosis and decreases the intraoperative awakeness and awareness incidence^[17,18]^. In our study, our BIS values during the operation were lower than these values. Especially the marked decrease in BIS values at the heart-lung pump period is explained by the prominent slowing effect of hypothermia in addition to the applied anesthesia on the brain's metabolism. None of our patients experienced clinical manifestations such as cerebral emboli, carotid stenosis or cerebrovascular accident, which may cause rapid decrease in the intraoperative brain blood flow that may result in a BIS value decrease. The course of the BIS values was generally consistent with the clinical data. In the literature, it is recommended to reevaluate the situation if the BIS values are inconsistent with the clinical course.

We used etomidate for anesthesia induction and propofol for maintenance treatment. For analgesia, fentanyl was used during the induction, and remifentanil for the maintenance treatment. With this application, adequate hypnosis and analgesia was provided. We obtained an almost similar decrease in BIS values with the induction in both groups. Remifentanil dose was slightly higher in the BIS value-known group, but this value was clinically not significant. The consciousness of the anesthesist about the BIS monitoring values of the patients didn't affect the drug doses during the anesthesia. Gan et al.^[19]^ in their study where they used propofol/alfentanil, detected a 13-23% decrease in hypnotic drug administration with the use of BIS.

To assess the intraoperative awareness, the patients were questioned after the operation about what they remembered from the time of operation. One patient from each group said that they had had a dream, and one patient in G2 heard a noise and felt that he was being carried from one place to another a few times. The BIS values were 50 and below in patients who had dreams and 73 at the end of the operation in the patient who had felt that he was being carried from one place to another, who was retreated with propofol and this value decreased to 31. In our study, a few patients experienced intraoperative awakeness and awareness in both groups and there was no statistically difference. This situation was explained with adequate depth of anesthesia given to our patients in both groups.

The awareness of the anesthesist about the BIS monitor values of the patients didn't make a difference that would affect the haemodynamic values. In our patients, haemodynamic stabilization was achieved with adequate anesthetic dose, according to the clinical data. Remifentanil has a minimal cardiac depressant effect except bradycardia which can be handled easily. Propofol is used frequently in cardiac surgery^[20,21]^. No prominent cardiac depressant effect was seen with etomidate, propofol and remifentanil, which were used in our study. Because the number of patients with haemodynamic instability was very low in our study, we think that the BIS monitoring doesn't have an additive effect on decreasing this risk.

As a result, BIS monitor values in cardiac surgery were consistent with the patients' clinical and haemodynamic values. The management should be done with clinical evaluation, haemodynamics and other monitorization methods. BIS monitoring is not an indispensable method, but when clinical haemodynamic values were perplex, it may be useful.

**Table:** 

Authors' roles & responsibilities	
HKK	Conception and design study; analysis and/or data interpretation; final approval of the manuscript
NS	Conception and design study; final approval of the manuscript
KO	Analysis and/or data interpretation; final approval of the manuscript
IG	Manuscript writing or critical review of its content; final approval of the manuscript
TAT	Manuscript writing or critical review of its content; final approval of the manuscript
